# Evaluation of Stability and In Vitro Security of Nanoemulsions Containing* Eucalyptus globulus* Oil

**DOI:** 10.1155/2017/2723418

**Published:** 2017-06-11

**Authors:** Samantha Nunes de Godoi, Priscilla Maciel Quatrin, Michele Rorato Sagrillo, Kátia Nascimento, Roger Wagner, Bruna Klein, Roberto Christ Vianna Santos, Aline Ferreira Ourique

**Affiliations:** ^1^Curso de Biomedicina, Centro Universitário Franciscano, Santa Maria, RS, Brazil; ^2^Programa de Pós-Graduação em Nanociências, Centro Universitário Franciscano, Santa Maria, RS, Brazil; ^3^Laboratório Escola de Análises Clínicas, Centro Universitário Franciscano, Santa Maria, RS, Brazil; ^4^Departamento de Tecnologia e Ciência dos Alimentos, Universidade Federal de Santa Maria, Santa Maria, RS, Brazil; ^5^Departamento de Microbiologia e Parasitologia, Universidade Federal de Santa Maria, Santa Maria, RS, Brazil

## Abstract

Essential oil of* Eucalyptus globulus* presents several pharmacological properties. However, their therapeutic efficacy may be affected by limitations due to several conditions, rendering it difficult to obtain stable and effective pharmaceutical formulations. The use of nanotechnology is an alternative to improve their characteristics aiming to ensure their stability and effectiveness. Furthermore, studies about the possible toxic effects of nanostructures are necessary to evaluate safety when the formulation comes into contact with human cells. Hence, in this paper, we evaluate for the first time the stability and in vitro cytogenotoxicity of nanoemulsions containing* Eucalyptus globulus* in peripheral blood mononuclear cells. As a result, the stability study found that the best condition for storage up to 90 days was refrigeration (4°C); it was the condition that best preserved the nanometric features. The content of the major compounds of oil was maintained after nanoencapsulation and preserved over time. In tests to evaluate the safety of this formulation, we can conclude that, at a low concentration (approximately 0.1%),* Eucalyptus globulus* nanoemulsion did not cause toxicity in peripheral blood mononuclear cells and also showed a protective effect in cells against possible damage when compared to oil in free form.

## 1. Introduction

According to the World Health Organization (WHO), a large part of the global population makes use of some type of plant to treat or improve the symptoms of pathological processes [1]. In addition, the use of natural medicine has gained prominence due to the absence of side effects [2].

Essential oils are natural compounds known for their medicinal properties and their use in the perfumery industry. In the environment, the essential oils have the function of protecting the plants against injuries caused by microorganisms [3]. Essential oils are products from plants, which has the vital function of causing a better adaptation of the plant in the environment where it is found; in addition, these oils can be obtained through several parts of these plants [4]. Currently, 10% of essential oils studied are used for industrial purposes, although thousands of species of essential oils are known [5].

In Brazil, the main species producing medicinal oil is* Eucalyptus globulus* [6]. Eucalyptus is a medicinal plant belonging to the Myrtle family [7], and although it is already a widely used species, all its medicinal benefits are not yet known; so it is important to study an essential oil from eucalyptus,* Eucalyptus globulus*, to better understand its pharmacological properties and possible disadvantages arising from its use [8, 9].

Among the disadvantages of this oil are susceptibility to volatilization, low aqueous solubility, and instability against the presence of oxygen and light, and in addition to impairing its therapeutic action, the production of effective formulations is disrupted [10].

A possibility of increasing the stability and efficacy of these compounds would be nanoencapsulation [11]. In the field of medicinal plants, the application of nanotechnology presents benefits such as increased apparent solubility, bioavailability, biocompatibility, and protection against physical and chemical degradation of the active principle, thereby inducing an increase in the pharmacological action and protection against toxicity [12]. Among the nanosystems, nanoemulsions are the best when used as carriers of essential oils, or their compounds, due to their high affinity provided in the formation of droplets, enhancing the internalization in biological membranes easily [13]. Nanoemulsions are stable oil droplets with an average size smaller than 100 nm formed by two immiscible phases stabilized by a surfactant. They may be obtained by different methods, depending on the desired structure and intended functionality [14]. Nanoemulsions have a transparent or translucent appearance and demonstrate greater stability when compared to conventional emulsions [15]. High energy to promote a shear rate is required; usually it is achieved through high-pressure homogenizers or ultrasonic generators [16]. The use of this type of nanosystem can increase the concentration of bioactive compounds in specific places of living organisms [17].

This nanosystem has been widely used in association with essential oils for the most diverse applications. Gomes et al. [18] showed the cytotoxic activity against tumor cell lines of two essential oils (*Drimys angustifolia* Miers and* D. brasiliensis* Miers) in nanoemulsions; Duarte et al. [19] evaluated the larvicidal activity of a nanoemulsion containing* Rosmarinus officinalis* essential oil against* Aedes aegypti*; and Lu et al. [17] proposed the use of citral nanoemulsions to improve the physicochemical stability of the compound in addition to enhancing its antimicrobial action.

It is possible to verify that nanoemulsions are systems widely used in association with essential oils for several areas, to improve the physical-chemical stability of the essential oil but also to maximize the action of this oil.

The production of nanoemulsions of* Eucalyptus (E. globulus)* has been reported in a few studies in the literature [20, 21]. These authors used concentrations of 6% and 10% of essential oil and successfully obtained the formulation containing this oil by ultrasonication technique.

As important as ensuring the efficacy of the formulation is to ascertain its safety; thus, the cell culture and tissue have been widely used for toxicity testing. These tests produce prior knowledge about the cytotoxicity of the substance when it comes into contact with the cells of living beings [22].

Therefore, this study aimed to prepare and characterize nanoemulsions containing 5% of* E. globulus* oil, to determine the best conditions for their storage and to evaluate the safety of these formulations while being in contact with healthy human cells (peripheral blood mononuclear cells).

## 2. Materials and Methods

### 2.1. Materials and Reagents

*E. globulus* oil was supplied by Ferquima (São Paulo, Brazil). Sorbitan monooleate (Span 80®) was supplied by Sigma-Aldrich (São Paulo, Brazil). Polysorbate 80 (Tween 80®) was supplied by LabSynth® (São Paulo, Brazil). Caprylic/capric triglyceride mixture was acquired from Alpha Química Ltda. (Porto Alegre, Brazil). Histopaque was supplied by Sigma-Aldrich (São Paulo, Brazil). Fetal Bovine Serum, RPMI 1640, and Penicillin/Streptomycin were supplied by Cultilab (São Paulo, Brazil). Dimethyl sulfoxide (DMSO) and trypan blue were supplied by Nuclear (São Paulo, Brazil).

### 2.2. Preparation of* E. globulus* Nanoemulsions

The nanoemulsions were prepared using the emulsification method under high agitation employing Ultra-Turrax® equipment with cooling. No organic solvent or rotary evaporator stage was used to prevent evaporation of volatile oil compound. The oil phase of the nanoemulsion consists of* E. globulus* oil (5%) and sorbitan monooleate (2%), while the aqueous phase comprises polysorbate 80 (2%) and ultrapure water. Both phases were previously solubilized with the aid of a magnetic stirrer, and then the oil phase was injected into the aqueous phase under agitation of 10,000 rpm. After the injection, the stirring was increased to 17,000 rpm and maintained for 1 hour. Blank nanoemulsions containing capric or caprylic acid triglyceride in place of* E. globulus* were also prepared in the same mode for comparative purposes. All formulations were produced in triplicate.

### 2.3. Characterization of the Essential Oil and Nanoemulsion Oil Content

The essential oil of* E. globulus* was characterized according to Amini et al. [23] with modifications in a Varian gas chromatograph, model Star 3400CX (CA, USA) equipped with a flame ionization detector (GC-FID). An aliquot of 1 *μ*L of the solutions was injected using a Varian autosampler model 8200 (CA, USA) in the chromatograph injection port under 250°C in split mode (1 : 20). The compounds were separated on a nonpolar capillary column of fused silica BPX 5 (25 m × 0.22 mm × 0.25 *μ*m) (SGE, Australia). Hydrogen was used as carrier gas at a constant pressure of 20 psi. The initial column temperature was adjusted at 40°C and held for 1 minute. Then, temperature increased at 1°C min^−1^ to 70°C and then to 220°C at 10°C min^−1^ and was kept under isothermal conditions for 3 minutes. The detector temperature was maintained at 250°C. A series of n-alkane homologues were analyzed under the same chromatographic conditions to calculate the linear retention index (LRI).

The qualitative analysis of the compounds was performed by a Shimadzu QP2010 Plus gas chromatograph coupled to a mass spectrometer (GC/MS, Shimadzu Corporation, Kyoto, Japan). For this analysis, the same chromatographic conditions described above were used and helium was used as the carrier gas. The detector was operated in electron impact ionization mode with 70 eV ionization energy and a mass scan range of 35–350 *m*/*z*. Analytes were identified based on comparison to the mass spectra in the library available at the National Institute of Standards and Technology (NIST) comparing the calculated linear retention indices with those available in the scientific literature. The percent relative amount of each identified compound was obtained from the peak area found using the FID.

The* E. globulus* oil had to be extracted from the nanoemulsions to analyze the oil content. For this, 100 *µ*L of the suspension was placed in microvial tubes and subjected to 50°C for 15 minutes in a dry block heating TE-O21 (Tecnal, Brazil). Then 1 mL of acetonitrile was added and the solution subjected to vortexing for 1 minute. Subsequently, the solution was centrifuged for 5 minutes at 10,000 rpm. Compared to pure commercial oil, used for the production of nanoemulsions, 5 *µ*L diluted in 1 mL of acetonitrile was subjected to chromatographic analysis under the same conditions previously described. These solutions were placed in injection vials where 1 *µ*L was injected into the chromatographic system of 1 *µ*L that was injected into the chromatographic system.

### 2.4. Characterization of Nanoemulsions

After the preparation, all formulations (produced in triplicate) were characterized by the following parameters: pH, average particle size, polydispersity index, zeta potential, oil content, and morphology.

The pH was measured directly in the formulations with the aid of a calibrated potentiometer (DM-22, Digimed®, Brazil). The dynamic light scattering technique using the Zetasizer equipment (Nano-ZS Zetasizer® model ZEN 3600, Malvern, United Kingdom) was used to determine the average particle size and polydispersity index (PDI). This technique measures the diffusion particles moving under Brownian motion and converts this measurement into size and size distribution of the formulations after dilution in ultrapure water previously filtered through a 0.45 *µ*m membrane (Sartorius RC 0.45 microns) [24].

The Doppler laser microelectrophoresis technique with Zetasizer equipment (Nano-ZS Zetasizer® model ZEN 3600, Malvern, United Kingdom) was used to evaluate the zeta potential, expressing the results in millivolts. An electric field is applied to a solution of molecules or a dispersion of particles, which will move at a velocity related to its zeta potential. This speed is measured by a laser interferometric technique (light scattering with phase analysis). In this regard, it is possible to determine the zeta potential through electrophoretic mobility calculation. The analyses were performed after dilution of the formulation in a 10 mM NaCl solution previously filtered through the 0.45 *µ*m membrane [24].

The oil content in the nanoemulsions was evaluated according to the methodology described above in Section 2.3.

According to Shi et al. [25], with modifications, the morphological analysis of the nanoemulsions containing* E. globulus* oil and blank nanoemulsions was evaluated using a transmission electron microscope at 80 kV (TEM, JEOL, JEM 1200 Exll, Japan). Samples were diluted in water (1 : 10 v/v) and dropped a drop on the grid. After 1 minute, the samples were dried with paper film and stained with uranyl acetate (2%) and were maintained in a vacuum desiccator until the microscopic evaluations which were analyzed at different magnifications.

### 2.5. Stability Study

The stability of formulations was done according to Isaac et al. [26] immediately after they were prepared. The batches were produced and the physicochemical parameters described above (except morphology) were evaluated under different storage conditions: room temperature (25°C), cooling (4°C), and climate chamber (40°C and 65 % humidity) at 0, 7, 15, 30, 60, and 90 days. For analysis, we compared these data to zero time (after preparation).

### 2.6. Cytogenotoxicity In Vitro Evaluation

Peripheral blood mononuclear cells were used to evaluate cytogenotoxicity. The peripheral blood samples were obtained from samples with no identification from the Laboratório Escola de Análises Clínicas of the Centro Universitário Franciscano, Santa Maria, RS, Brazil, approved by the Ethics Committee for Research on Human Beings of Centro Universitário Franciscano (Protocol number CAAE: 31211214.4.0000.5306). The samples were obtained by venipuncture using heparin-like Vacutainer® tubes. The separation of mononuclear cells from peripheral blood (PBMC) by density gradient was performed (Histopaque®-1077) by centrifugation, and the concentration of 2 × 10^5^ cells was obtained by counting in a Neubauer chamber with trypan blue 0.4% staining.

From the* E. globulus* oil concentration in this formulation (5%), three dilutions were standardized in pure RPMI (0.1%/0.2%/0.3%) to be tested for genotoxicity in our nanoencapsulated essential oil, free oil, and blank formulation.

To verify the toxic effects of compound on cell viability and DNA damage, an experimental protocol was conducted similar to that described by Wilms et al. [27]. Three different groups were tested in cells: (a) nanoemulsion containing the* E. globulus *oil (NEO); (b)* E. globulus* free oil (FO); (c) blank nanoemulsion (BNE), composed of medium chain triglycerides replacing* E. globulus *oil in the formulation, being tested for the purpose of evaluating possible toxicity from the nanostructure composition. In addition, the controls used for the tests were a negative control (C−) consisting of culture medium containing the PBMC and a positive control (C+) containing culture medium and PBMC added 100 mM hydrogen peroxide (H_2_O_2_).

#### 2.6.1. Cell Viability Assay

Cell viability was analyzed according to Mosmann [28]. The cytotoxic activity in peripheral blood mononuclear cells was evaluated by the colorimetric method whose principle is based on reduction of 3-(4,5-dimethyl thiazol-2-yl)-2,5-diphenyl tetrazolium bromide (MTT) in a dark purple MTT-formazan through mitochondrial succinate dehydrogenase tetrazolium enzyme activity. As the conversion occurs only in viable cells, the decreased absorbance of the test compared to negative controls indicates cell death. The experiment was performed in triplicate in a 96-well plate with samples and controls, negative and positive as described above. The plate was incubated at 37°C with 5% CO_2_ during 72 hours. Then the sample absorbances were determined in a spectrophotometer at 570 nm. The results were expressed as percentage of control.

#### 2.6.2. Lipid Peroxidation Measurement

The determination of lipid peroxidation was assessed by determining the thiobarbituric acid reactive species according to the method described by Ohkawa et al. [29] and modified by Carrera-Rotllan and Estrada-Garcia [30]. After 72 hours of incubation, an aliquot of each treatment was mixed with a reaction medium containing 2-thiobarbituric acid (TBA) (0.8%) and incubated at 95°C for 1 hour. The absorbance was measured at a wavelength of 532 nm in a spectrophotometer and the results expressed as moles of malondialdehyde (MDA).

#### 2.6.3. Protein Carbonylation Analysis

Oxidative damage of proteins was measured by determining the formation of carbonyl groups based on the reaction with dinitrophenylhydrazine as previously described by Levine et al. [31]. After homogenization, the carbonyl buffer samples (120 mm KCl, 30 mm KH_2_PO_4_) were centrifuged at 7000*g* for 15 minutes at 4°C and lysed in 20% trichloroacetic acid (TCA). After lysing, the samples were centrifuged again at 14,000*g* for 5 minutes. The pellet was resuspended in 100 *µ*L of 0.2 M NaOH and the samples were incubated for 1 hour at room temperature with 2 M HCl (1 : 3) added to 10 mM 2,4-dinitrophenylhydrazine (DNPH). After the incubation period, 100 *µ*L of trichloroacetic acid (TCA) 20% was added and samples were centrifuged at 14,000 ×g for 3 minutes. The pellet was washed with 500 *µ*L of ethanol-ethylacetate. Samples were incubated for 30 minutes at 60°C and centrifuged again at 14,000*g* for 3 minutes. The formation of carbonyl content was determined spectrophotometrically at 370 nm.

#### 2.6.4. Single Cell Gel Electrophoresis Assay: Comet Assay

The comet assay was performed according to Singh et al. [32] and modified by García et al. [33]. This test is highly sensitive and allows quantifying the levels of single-strand DNA breaks. On a glass slide covered previously with a 1.5% agarose layer, the mononuclear cells from peripheral blood were deposited and suspended in low-melting point agarose (Low Melting). The material was immersed in lysis solution (89 mL lysing solution to 10 mL of dimethyl sulfoxide and 1 mL of Triton X-100) for the removal of membranes and cytoplasm. Subsequently, the slides were incubated in alkaline electrophoresis buffer (300 mM NaOH and 1 mM EDTA in distilled water) and subjected to electrophoresis for 30 minutes at 25 V and 300 mA. Subsequently, the neutralization process was performed, fixation and staining by which genetic material can be analyzed.

Damage index calculation formula is DI = (*D*_1_ + *D*_2_ + *D*_3_ + *D*_4_)/100.

#### 2.6.5. Hemolysis Evaluation

According to Pequeno and Soto-blanco [34], the hemolytic activity of all treatments was measured in a spectrophotometer using a hemoglobin release assay. Summarily, defibrillator erythrocytes were washed three times with PBS 1x and centrifuged for 15 minutes at 9,000*g*. After washings, a pool of erythrocytes was concentrated in a 10 mL tube. After this pool, 400 *µ*L of this procedure was removed and resuspended in 1 mL PBS 1x. Subsequently, the cells were treated for 1 hour at 37°C and then centrifuged at 1,000 rpm for 10 minutes. Aliquots of the supernatant were transferred to a microcentrifuge tube, where the release of hemoglobin was monitored using a PD-Reader microplate reader (Thermoplate®, China), measuring the absorbance at 409 nm. Percentage of hemolysis was calculated as (AT − AC)/(AC+ − AC−) × 100, where AT is the absorbance of the supernatant treated, AC− is the absorbance of the supernatant from control, cells treated with PBS, and AC+ is the absorbance of the treatment with hydrogen peroxide, which proves positive hemolytic activity.

### 2.7. Statistical Analysis

The characterization data and the study data “in vitro” were analyzed using Graphpad Prism 5.0 software followed by one-way ANOVA with* Dunnett's* post hoc test. Values of *p* < 0.05 were considered statistically significant for both tests.

## 3. Results and Discussion

### 3.1. Characterization of the Essential Oil and Oil Content in the Nanoemulsions

The chemical components of* E. globulus* oil are shown in Table 1. Its main components are 1,8-cineole (75.7%); p-cymene (7.5%); alpha-pinene (7.3%); and limonene (6.4%). According to Vitti and Brito [35], the main component of* E. globulus* oil is the compound 1,8-cineole with an average concentration of 80%. This composition corroborates our results of the oil composition (Table 1) by gas chromatography. Regarding oil content in nanoemulsions, the authors reported that the nanoemulsion preserved the percentage of the main oil compound, 1,8-cineole, for 90 days. This shows that the nanostructuring proposed in this study did not damage the oil composition.

### 3.2. Characterization of Nanoemulsion and Stability Study

After preparation, the nanoemulsions presented adequate nanometric properties as shown by Codevilla et al. [36] and Schaffazick et al. [37], with mean particle diameter and polydispersity index around 70 nm and 0.2, respectively, a zeta potential of approximately −9 mV, and acid pH (Table 2).

**Figure 1:**
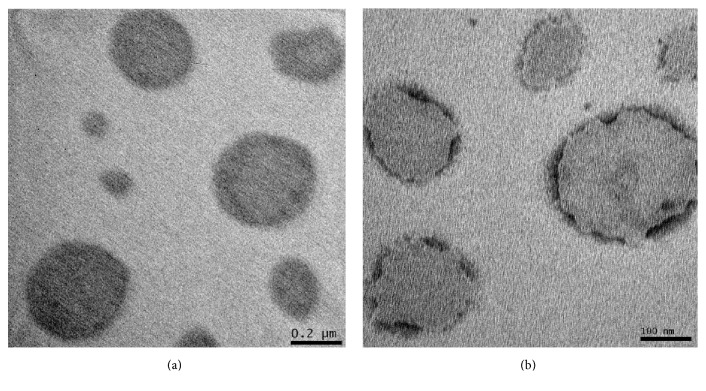
Transmission electron microscopy images of nanoemulsion of* Eucalyptus globulus* oil (a) and blank nanoemulsion (b) [bar = 200 nm (100,000x)].

Scanning electron microscopy (SEM) and transmission (TEM) have long been used to obtain information about the shape and size of nanoparticles [37]. In this study, the morphological analysis of nanoemulsions containing* E. globulus* by transmission electron microscopy showed spherical particles (Figure 1(a)) and (Figure 1(b)) representing blank nanoemulsions, where the diameters were similar to those found in dynamic light scattering analysis, as demonstrated by Lee et al. [38], respecting the peculiarities of each technique.

According to Schaffazick et al. [37], the industrial advantage of nanoparticles dispersed in aqueous medium may present limitations, due to issues of poor physicochemical stability in storage for extended periods. Based on this, the authors observe that the formulation stored in a climate chamber after 7 days had a mean diameter of approximately 258 nm; after 15 days, this formulation showed a polydispersity index of 0.3. After being stored for 60 days, phase separation occurred, which made it impossible to read the parameters. It was then possible to verify instability in the formulation when stored at elevated temperatures (40°C). It is suggested that a possible reason for instability of the* E. globulus *in this condition has been the formulation of an oxidative process caused by the high temperature at which it is stored.

In the formulation stored at room temperature (25°C) within 90 days, an increased size and zeta potential to about 152.47 nm and −18.87 mV, respectively, were observed, and the polydispersity index was 0.07, and the pH was close to 3.

The formulation kept under refrigeration retained its nanometric characteristics 90 days after preparation, while keeping its average particle size of approximately 75 nm, polydispersion index of 0.2, the zeta potential of −10.4 mV, and acidic pH (around 4.2). This study showed evidence that cooling is the best form of storage for nanoemulsions.

### 3.3. Evaluation of Cytogenotoxic Effects

As described by Maluf and Riegel [39], peripheral blood mononuclear cells (PBMC) have been used for years as biological markers of cytogenotoxic effects. Because they are abundant in the bloodstream, these cells can be exposed to any mutagen and have the ability to demonstrate recent damage. As the PBMC grow, they provide a very promising in vitro model for various studies, and the usefulness of this cell line is mentioned in cytogenotoxicity studies.

#### 3.3.1. Cell Viability Assay

The human structure suffers continuous action of reactive oxygen species (ROS) and reactive nitrogen species (RNS) produced in inflammatory processes arising from biological or nutritional changes. The main ROS are the superoxide anion that is catalyzed by catalase and/or GPx to hydrogen peroxide and plays an important role in oxidative stress, having the ability to cross cell membranes and produce hydroxyl radicals [40]. Thus it is highly toxic to the cells [41].

In this study, hydrogen peroxide (H_2_O_2_) was used as a positive control indicating cell death due to its high performance as a reactive species; therefore, values less than the positive control are not viable and cell values greater than the negative control indicate cell proliferation.

**Figure 2:**
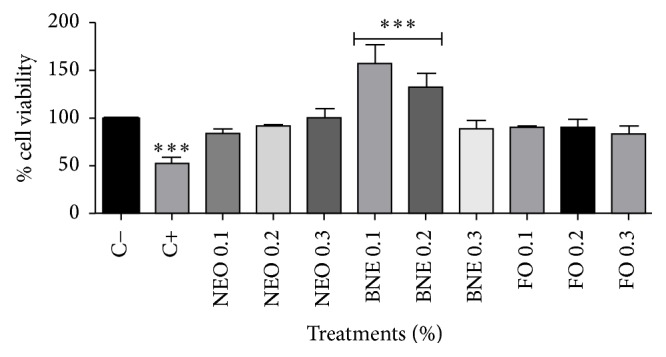
MTT assay after 72 h of incubation. Results expressed as percentage of the negative control (100%). Negative control (C−): cells in culture medium; positive control (C+): cells with H_2_O_2_. Data are expressed as mean ± standard deviation (SD). Analyses were performed by variance (ANOVA) of one way, followed by* Dunnett's *test. Values with *p* < 0.05 were considered statistically significant. ^*∗*^*p* < 0.05, ^*∗∗*^*p* < 0.01, and ^*∗∗∗*^*p* < 0.001.

Figure 2 shows the results of the MTT assay on PBMC after 72 hours of treatment. This analysis demonstrated that none of the test groups compared to negative control caused cell death. These results showed that neither the free* E. globulus* oil nor* E. globulus* nanoemulsion and blank formulation caused damaged to the cell mitochondria, suggesting that they do not present cytotoxic effects.

#### 3.3.2. Lipid Peroxidation Measurement

**Figure 3:**
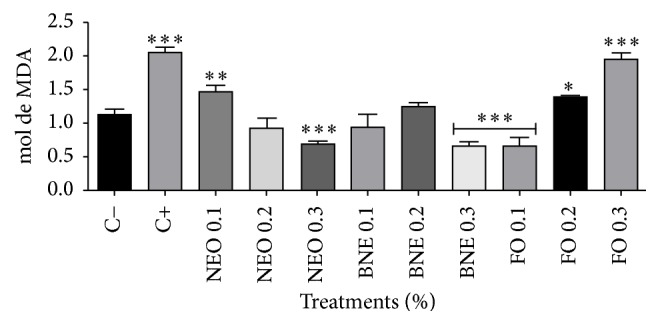
TBARS assay with 72 hours of incubation. Results expressed as percentage of the negative control (100%). Negative control (C−): cells in culture medium; positive control (C+): cells with H_2_O_2_. Data are expressed as mean ± standard deviation (SD). Analyses were performed by variance (ANOVA) of one way, followed by* Dunnett's *test. Values with *p* < 0.05 were considered statistically significant. ^*∗*^*p* < 0.05, ^*∗∗*^*p* < 0.01, and ^*∗∗∗*^*p* < 0.001.

Lipid peroxidation in biological processes can occur in several ways including nonenzymatic means, where the mechanism involves reactive oxygen species, free radicals, and other metals. All oxidative processes trigger changes in physical and chemical properties of the membrane, altering the fluidity and permeability by spreading the intracellular fluid and risk of rupture of cell membranes and organelles, resulting in cell death [42]. The product of this lipid peroxidation is malondialdehyde which is a reactive aldehyde used widely as a biomarker [43]. In Figure 3, we can observe the levels of TBARS in PBMC after 72 hours of treatment with NEO, BNE, and FO at three concentrations. The results showed that the free* E. globulus* oil (FO) demonstrated a dose-dependent toxicity profile and the production of thiobarbituric acid reactive species increased as the oil concentration increased; we can highlight the concentration of 0.3% which was equal to the negative control.

When PBMC came into contact with* E. globulus* nanoemulsions (NEO), the profile observed was the contrary of that in the graph shown above, because when the concentration of NEO increased, the cells were protected by oil nanoencapsulation. And the BNE did not induce cell oxidation, proving the nontoxicity of the formulation proposed in this paper.

#### 3.3.3. Protein Carbonylation Analysis

Carbonylation can alter the conformation and activity of proteins and trigger the formation of protein aggregates [44]. According to the descriptions by Nyström [45], the task of identifying the factors that trigger carbonylation has been difficult, but there are many possibilities including the reduction of the antioxidant defense process and an increase in the generation of reactive oxygen species (ROS) reducing the ability to remove oxidized proteins or increasing the sensitivity of proteins to oxidative attack.

**Figure 4:**
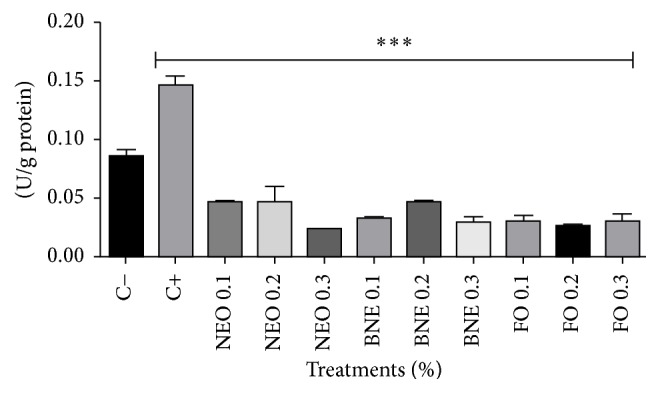
Protein carbonylation assay with 72 hours of incubation. Results expressed as percentage of the negative control (100%). Negative control (C−): cells in culture medium; positive control (C+): cells with H_2_O_2_. Data are expressed as mean ± standard deviation (SD). Analyses were performed by variance (ANOVA) of one way, followed by* Dunnett's *test. Values with *p* < 0.05 were considered statistically significant. ^*∗*^*p* < 0.05, ^*∗∗*^*p* < 0.01, and ^*∗∗∗*^*p* < 0.001.

Figure 4 shows the evaluation of protein carbonylation induction after 72 hours of exposure to the treatment conditions. The results show that free* E. globulus* oil, nanoemulsion from* E. globulus*, and blank nanoemulsion did not cause protein damage to the cells compared to negative control.

#### 3.3.4. Single Cell Gel Electrophoresis Assay: Comet Assay

As described by Brianezi [46], the study of comets is the degree of DNA fragmentation and its migration by microelectrophoresis. Currently, this method is a widely used genotoxicity test of industrial, pharmaceutical, and agrochemical products for damage and DNA repair in individual cells. It is relatively easy to implement, is fast, is of a lower cost, and is safe.

Each slide was analyzed under a light microscope and the cells were classified according to the image, based on four classes of damage proposed by García and coworkers [33] and illustrated by Fronza and collaborators [47], ranging from 0 (no damage) to 4 (maximum injury), but also including cellular apoptosis rating.

**Figure 5:**
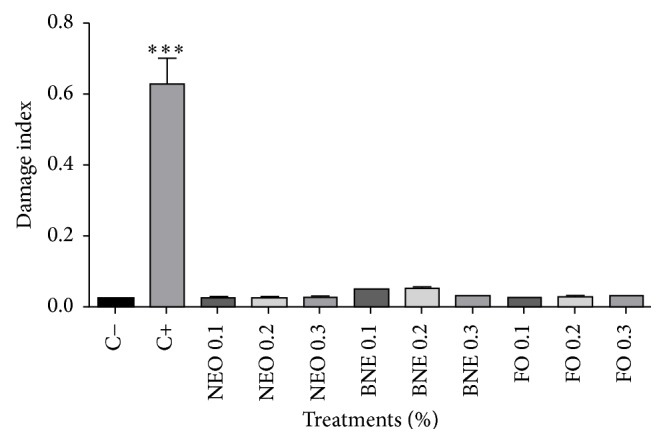
Comet assay with 72 hours of incubation. Results expressed as percentage of the negative control (100%). Negative control (C−): cells in culture medium; positive control (C+): cells with H_2_O_2_. Data are expressed as mean ± standard deviation (SD). Analyses were performed by variance (ANOVA) of one way, followed by* Dunnett's *test. Values with *p* < 0.05 were considered statistically significant. ^*∗*^*p* < 0.05, ^*∗∗*^*p* < 0.01, and ^*∗∗∗*^*p* < 0.001.

Based on Figure 5, we compare the results obtained with the negative control DNA damage; we can say that the nanoemulsion containing* E. globulus* oil, free oil, and blank nanoemulsion did not cause breakage or damage to the DNA.

#### 3.3.5. Hemolysis Evaluation

**Figure 6:**
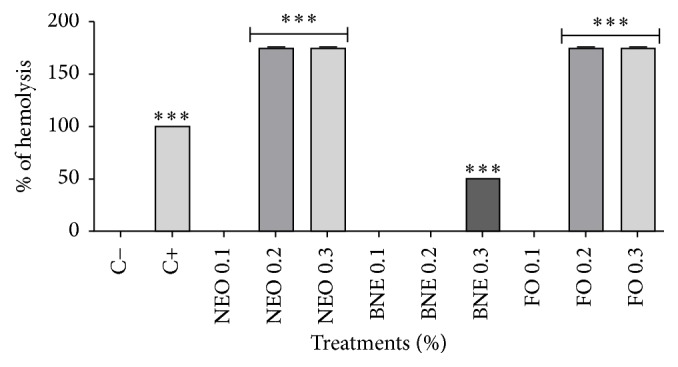
Hemolysis assay after 1 hour of incubation. Results expressed as percentage of the negative control (100%). Negative control (C−): cells in culture medium; positive control (C+): cells with H_2_O_2_. Data are expressed as mean ± standard deviation (SD). Analyses were performed by variance (ANOVA) of one way, followed by* Dunnett's *test. Values with *p* < 0.05 were considered statistically significant. ^*∗*^*p* < 0.05, ^*∗∗*^*p* < 0.01, and ^*∗∗∗*^*p* < 0.001.

Based on Figure 6, we found that the nanoemulsion at a concentration of 0.1% oil had a better protective effect on the cells by preventing cell destruction, a process called hemolysis. Likewise, the behavior of the BNE and the FO (both 0.1%) did not cause cellular destruction at the lowest concentration showing that the formulation and oil are nontoxic in this case.

Already at a concentration of 0.2%, a different situation was evidenced, in which the NEO and FO caused hemolysis. It is assumed that this may have been caused by the action of some component present in the* E. globulus* oil, as compared to the BNE and the FO or NEO. We found that the formulation without oil (BNE) at this concentration did not induce cell lysis. According to Bruxel et al. [48], the use of nonionic surfactants from the group of poloxamer and polyoxyethylene sorbitans (Tweens) has shown a favorable in association with phospholipids, as they lead to the formation of compact mixed films, conferring greater stability to the formulation. However, hemolytic reactions and changes in droplet diameter of nanoemulsions stabilized with Tween 80 can limit their use alone, which was verified at the concentration of 0.3% also for the NEB.

We conclude that the safety of the formulation is minimized when the oil is found at higher concentrations causing toxicity against red blood cells triggering hemolytic action. This behavior can be attributed to any compound present in the oil, as demonstrated in the study by Mendanha et al. [49], which showed hemolytic action in erythrocytes when exposed to terpene 1-8 cineol at high concentrations, corroborating our results since this is the main component of* E. globulus* oil. It is also assumed that this may be due to limitations of the technique, such as the short analysis time while this method is being carried out, improperly performed blood collection, or a prior hemolytic condition of the patient. Based on the above discussion, further studies are suggested to investigate the real cause of the hemolytic process triggered in the presence of nanostructures.

## 4. Conclusion

The present study performed a successful nanoencapsulation of essential oil of* Eucalyptus (E. globulus)* at a concentration of 5% through nanoemulsions, which, after evaluation, showed adequate nanometric properties according to the literature.

It is noteworthy that, for the methodology of nanoemulsion, no organic solvent was used or it was employed using a rotary evaporator stage. Furthermore, there was temperature control throughout the process, thus avoiding the volatilization of oil or its structural components, minimizing the chances of an ineffective encapsulation process.

We also demonstrated a prolonged durability profile when the nanoemulsions were stored under refrigeration (4°C), storage being possible for 90 days without any loss of stability. Nanoemulsions produced showed appropriate morphology and preserved the same amounts of the major component of the essential oil in the nanostructure within 90 days.

Through the study of cytogenotoxicity in PBMC, we can say that* E. globulus* nanoemulsion did not cause cell death; it showed a protective effect on cells when exposed to species of lipid peroxidation; it also did not provide protein carbonyls or cause damage to the DNA of the cells tested; however, it should be noted that the* E. globulus* nanoemulsion at high concentrations may trigger lysis of erythrocytes. Based on these results, we can say that 0.1% is the safest concentration for the use of* Eucalyptus globulus* nanoemulsions in human cells.

## Figures and Tables

**Table 1:** Free *E. globulus* oil composition and oil composition in nanoemulsion.

Compound	Normalization (% free oil)	Normalization^*∗*^ ± SD			
		(% oil in nanoemulsion)			
		After preparation	Day 30	Day 60	Day 90
alpha-Pinene	7.39	5.55 ± 0.13	5.39 ± 0.59	5.28 ± 0.84	5.39 ± 1.73
beta-Pinene	0.38	0.29 ± 0.00	0.30 ± 0.02	0.28 ± 0.04	0.29 ± 0.04
beta-Myrcene	0.41	0.32 ± 0.01	0.32 ± 0.02	0.25 ± 0.07	0.34 ± 0.05
alpha-Phellandrene	0.19	0.00 ± 0.00	0.00 ± 0.00	0.00 ± 0.00	0.03 ± 0.05
p-Cymene	7.55	8.14 ± 0.09	8.33 ± 0.27	8.37 ± 0.21	7.89 ± 0.41
Limonene	6.41	6.06 ± 0.33	5.94 ± 0.04	5.59 ± 0.50	3.96 ± 0.38
*1-8-Cineole*	*75.78*	*77.30 ± 0.34*	*78.09 ± 0.45*	*78.54 ± 1.23*	*81.16 ± 0.71*
gamma-Terpinene	1.11	0.29 ± 0.03	0.20 ± 0.15	0.17 ± 0.00	0.24 ± 0.32
trans-Pinocarveol	0.11	0.14 ± 0.01	0.19 ± 0.01	0.13 ± 0.01	0.10 ± 0.09
4-Terpineol	0.16	0.23 ± 0.01	0.22 ± 0.00	0.22 ± 0.01	0.18 ± 0.02
alpha-Terpineol	0.41	0.51 ± 0.04	0.51 ± 0.02	0.53 ± 0.04	0.40 ± 0.09
Aromadendrene	0.03	0.04 ± 0.00	0.04 ± 0.00	0.16 ± 0.06	0.03 ± 0.01

^*∗*^The analysis was performed in triplicate (*n* = 3).

**Table 2:** Stability of the nanoemulsions containing *Eucalyptus globulus* oil in 0–90-day periods.

Time	Conditions	Size	PDI ± SD	Zeta potential	pH ± SD
		(nm) ± SD		(mV) ± SD	
After preparation	—	75.57 ± 14.73	0.22 ± 0.04	−9.42 ± 1.15	4.68 ± 0.05

7 days	RT	75.74 ± 5.85	0.25 ± 0.03	−15.83 ± 1.62^**∗∗**^	4.26 ± 0.08^**∗∗**^
	RE	73.81 ± 8.35	0.23 ± 0.03	−7.97 ± 2.10	4.55 ± 0.06^**∗**^
	CC	257.93 ± 66.86	0.26 ± 0.08	−10.50 ± 2.24	3.25 ± 0.04^**∗∗∗**^

15 days	RT	74.23 ± 6.07	0.24 ± 0.03	−19.5 ± 2.02^**∗∗∗**^	4.93 ± 0.08^**∗**^
	RE	73.71 ± 14.80	0.26 ± 0.05	−9.26 ± 0.71	4,33 ± 0.04^**∗∗∗**^
	CC	248.93 ± 97.94	0.35 ± 0.12	−17.53 ± 4.09^**∗∗**^	4.12 ± 0.05^**∗∗∗**^

30 days	RT	76.92 ± 0.45	0.24 ± 0.07	−14.03 ± 3.30	3.66 ± 0.13^**∗∗∗**^
	RE	80.03 ± 17.55	0.32 ± 0.10	−11.63 ± 3.69	4.24 ± 0.14^**∗∗∗**^
	CC	488.93 ± 147.76^**∗∗**^	0.56 ± 0.09^**∗∗**^	−22.27 ± 2.73^**∗∗**^	2.91 ± 0.07^**∗∗∗**^

60 days	RT	83.37 ± 3.87	0.18 ± 0.04	−12.60 ± 0.29	3.14 ± 0.12^**∗∗∗**^
	RE	76.04 ± 6.03	0.25 ± 0.03	−11.57 ± 4.15	4.08 ± 0.05^**∗∗∗**^
	CC	486.20 ± 92.48^**∗∗**^	0.53 ± 0.09^**∗**^	−23.37 ± 2.17^**∗∗∗**^	2.72 ± 0.07^**∗∗∗**^

90 days	RT	152.47 ± 38.81^**∗∗**^	0.07 ± 0.06^**∗**^	−18.87 ± 0.31^**∗∗∗**^	2.99 ± 0.11^**∗∗∗**^
	RE	74.97 ± 8.36	0.24 ± 0.02	−10.40 ± 2.61	4.21 ± 0.02^**∗∗∗**^
	CC	—	—	—	—

Storage conditions: RT: room temperature (25°C); RE: refrigeration (4°C); CC: climatic chamber (40°C and 65% humidity). The analyses were carried out by variance (ANOVA) of one way, followed by *Dunnett's* test. Values of *p* < 0.05 were considered statistically significant. ^*∗*^*p* < 0.05, ^*∗∗*^*p* < 0.01, and ^*∗∗∗*^*p* < 0.001.
